# Enhanced contrast synchrotron X-ray microtomography for describing skeleton-associated soft tissue defects in zebrafish mutants

**DOI:** 10.3389/fendo.2023.1108916

**Published:** 2023-03-06

**Authors:** Jake Leyhr, Sophie Sanchez, Kathleen N. Dollman, Paul Tafforeau, Tatjana Haitina

**Affiliations:** ^1^ Department of Organismal Biology, Uppsala University, Uppsala, Sweden; ^2^ European Synchrotron Radiation Facility, Grenoble, France

**Keywords:** iodine staining, propagation phase-contrast synchrotron microtomography, virtual histology, zebrafish mutant, 3D segmentation, inner ear, Weberian apparatus, nkx3.2

## Abstract

Detailed histological analyses are desirable for zebrafish mutants that are models for human skeletal diseases, but traditional histological techniques are limited to two-dimensional thin sections with orientations highly dependent on careful sample preparation. On the other hand, techniques that provide three-dimensional (3D) datasets including µCT scanning are typically limited to visualizing the bony skeleton and lack histological resolution. We combined diffusible iodine-based contrast enhancement (DICE) and propagation phase-contrast synchrotron radiation micro-computed tomography (PPC-SRµCT) to image late larval and juvenile zebrafish, obtaining high-quality 3D virtual histology datasets of the mineralized skeleton and surrounding soft tissues. To demonstrate this technique, we used virtual histological thin sections and 3D segmentation to qualitatively and quantitatively compare wild-type zebrafish and *nkx3.2* ^-/-^ mutants to characterize novel soft-tissue phenotypes in the muscles and tendons of the jaw and ligaments of the Weberian apparatus, as well as the sinus perilymphaticus associated with the inner ear. We could observe disrupted fiber organization and tendons of the adductor mandibulae and protractor hyoideus muscles associated with the jaws, and show that despite this, the overall muscle volumes appeared unaffected. Ligaments associated with the malformed Weberian ossicles were mostly absent in *nkx3.2* ^-/-^ mutants, and the sinus perilymphaticus was severely constricted or absent as a result of the fused exoccipital and basioccipital elements. These soft-tissue phenotypes have implications for the physiology of *nkx3.2* ^-/-^ zebrafish, and demonstrate the promise of DICE-PPC-SRµCT for histopathological investigations of bone-associated soft tissues in small-fish skeletal disease models and developmental studies more broadly.

## Introduction

Skeletal diseases primarily affect bones and are genetically determined or caused by developmental abnormalities (1). In addition to bones, joints and cartilages can also be affected in skeletal diseases as endochondral bones are formed on the basis of cartilage anlagen during the development (2). Furthermore, some bone disorders like *osteogenesis imperfecta* can also display weakened muscle phenotypes (3) and in several diseases the associated tissues like bone-to-bone connecting ligaments and muscle-to-bone connecting tendons are also affected (4).

Genetic mutations that cause skeletal diseases can be linked to the genes that function as building blocks of cartilage and/or bone, but also as contributors to the structural and mechanical properties of the attached muscles or other associated tissues (e.g. collagen type I) (4). In addition, the absence, reduction, or deformation of skeletal components can also affect the associated tissue that requires direct interaction with the skeletal component during development. This interaction can be primarily mechanical, such as between the brain and skull in craniosynostosis (5), but can also involve inter-tissue crosstalk *via* signaling molecules (6). Furthermore, connective tissue diseases linked to bone metabolism and its endocrine function can trigger the immune system and manifest phenotypes in musculoskeletal, cardiovascular, respiratory, and ocular systems (7).

Together with the mouse, the zebrafish (*Danio rerio*) is an important and well-established model organism for human skeletal diseases (8, 9). There is at least one zebrafish orthologue for ~80% of human genes involved in disease (10), and many genes are conserved in their function, displaying overlapping expression patterns during skeletal development in zebrafish and human (11). This knowledge facilitated the generation of zebrafish gene mutants that was further accelerated by the development of CRISPR/Cas9 genome editing knock-out, knock-in, and genomic deletion approaches (12, 13), leading to a dramatic expansion of available zebrafish gene mutants in recent years. As a large number of mutants lack an easily detectable phenotype, a thorough examination approach at a histological level is needed in order to identify and link the mutant phenotypes to the human pathologies, and to understand their mechanistic basis (14–16). However, while traditional histology can provide extremely detailed information (17), it is constrained by slice orientation and physical section thickness that provides poor z-resolution, limiting attempts to understand morphological structures in 3D.

Skeletal phenotypes are most often described by investigating mutants in a transgenic background of fluorescently-labeled skeletal cells or by applying fluorescent and/or chromogenic stains of cartilage and bone, however detailed characterization in 3D by using these methods is often very limited. Confocal, light-sheet, and other advanced 3D optical microscopy techniques rely on optically transparent specimens and specific labelling of cells/tissues intended to be visualized, both of which can require complex preparation protocols and imaging setups when the specimens in question are relatively large and pigmented. As the skeleton mineralizes and becomes X-ray dense, imaging by X-ray micro-computed tomography (µCT) has proven to be a simple and powerful technique for the characterization of adult skeletal structures in 3D, useful in describing normal development and mutant phenotypes (e.g. 18–20).

3D phenotypic characterization using µCT is optimized for the visualization of highly dense mineralized structures while other tissues are barely detectable, limiting our understanding of how skeletal phenotypes interact with the surrounding soft tissues. By using tissue contrasting agents such as phosphotungstic acid (PTA) or iodine (I_2_E, I_2_M, or I_2_KI), the soft tissue density can be enhanced and visualized together with the mineralized structures by X-ray imaging (21–23). This enhances contrast by increasing the attenuation of X-rays passing through the soft tissues. The use of propagation phase-contrast imaging can complement this method to obtain sharp images at submicron resolution (24). This technique relies on the detection of changes in the phase of a coherent X-ray beam caused by slight differences in the thickness and refractive index of tissues to enhance the contrast at the edges of microstructural boundaries (25–27). The best source of coherent X-rays are synchrotron light sources (28), which additionally provide advantages over conventional µCT systems by reducing imaging times for an improved scan quality (with reduced to no beam hardening) and higher spatial resolution (29, 30).

*Spondylo-megaepiphyseal-metaphyseal dysplasia* is caused by homozygous mutations in human NK 3 Homeobox 2 (*NKX3.2*) gene (31, 32). Recently, the skeletal phenotypes associated with homozygous knockout mutants of the *nkx3.2* gene in zebrafish were characterized in detail using several techniques including X-ray microcomputed tomography (33, 34). These phenotypes included the fused jaw joint leading to an externally visible and permanently open jaw, loss of vertebral parapophyses, and defects in the occiput and Weberian apparatus (34).

Here, we combined diffusible iodine-based contrast enhancement (DICE) and propagation phase-contrast synchrotron radiation micro-computed tomography (PPC-SRµCT) to image late larval and juvenile *nkx3.2* ^-/-^ mutant and wild-type zebrafish. We obtained high-quality high-resolution 3D virtual histology datasets and characterized novel soft-tissue phenotypes in the muscles, ligaments, and inner ear that are associated with previously described skeletal defects.

## Materials and methods

### Ethical statement

All animal experimental procedures were approved by the local ethics committee for animal research in Uppsala, Sweden (permit number 5.8.18-18096/2019). All procedures for the experiments were performed in accordance with the animal welfare guidelines of the Swedish National Board for Laboratory Animals.

### µCT scanning

µCT data from 1, 2, and 3 months post fertilization (mpf) wild-type zebrafish were obtained as part of a previous study (34). Fixed specimens were embedded in 1% agarose and scanned in 2mL microcentrifuge tubes using a SkyScan 1172 (Bruker microCT, Belgium) at a voltage of 60kV, a current of 167µA, and an isotropic voxel size of 5.43µm.

### Iodine staining

Zebrafish homozygous for the previously published *nkx3.2^uu2803^* mutant allele (34) together with wild-type AB and *nkx3.2* ^+/+^ zebrafish were euthanized at 1, 2, and 3mpf with an overdose of MS-222 (300mg/L) and fixed in 4% paraformaldehyde. Specimens were rinsed in 1x phosphate-buffered saline and gradually dehydrated in 25%, 50%, 75% and 100% ethanol. Staining with iodine was performed overnight in 2mL microcentrifuge tubes (one fish per tube) with 1.5mL of 1% iodine solution in 100% ethanol according to the previously published protocol for I_2_E staining (21). After staining specimens were transferred to 96% ethanol for scanning.

### Synchrotron scanning

Iodine-stained zebrafish specimens were imaged using PPC-SRμCT (29) at beamline BM05 of the European Synchrotron Radiation Facility – Extremely Brilliant Source (ESRF-EBS) in France. They were imaged with two isotropic voxel sizes: 3µm and 0.727µm. The propagation distances were selected to optimize phase retrieval with a given photon energy (keV) and detector pixel size within the near-field Fresnel region (25, 35). The detector exposure times were optimized for the maximum dynamic range of the 16-bit image without saturation of the detector. The number of projections was optimized for high resolution and phase retrieval on the given detector pixel size (2048 x 2048) and the scanning mode (half-acquisition mode).

#### Medium-resolution configuration: 3µm voxel size

The specimens were imaged with a PCO Edge CLHS 4.2 camera using a magnifying optic based on a Canon 68mm F/D 2.8 Super Macro lens coupled to a 250µm-thick LuAG:Ce scintillator. The protocol of Hierarchical Phase-Contrast Tomography (HiP-CT) developed by Walsh et al. (36) was used to optimize the overall contrast of the data as well as the signal-to-noise ratio. The beam was filtered with 2.3mm of aluminum and 8 5mm bars of silicon dioxide. The resulting detected average energy was 78keV. In order to enlarge the lateral field of view, the samples were imaged in half acquisition, i.e. over 360 degrees around the center of rotation shifted by 900 pixels to the right side of the field of view. A total of 6000 projections, resulting from an accumulation of 4 sub-frames of 10ms each, were taken over 360 degrees. The specimens were placed at a propagation distance of 1.45m from the detector in order to maximize the phase-contrast effect. The relatively high energy results in the bones and the stained tissues having quite similar phase properties, making it possible to use a relatively simple approach for phase imaging.

#### High-resolution configuration: 0.727µm voxel size

The specimens were imaged with a PCO Edge 4.2 camera mounted on the Twinmic white-beam microscope system (Optique Peter) using a Mitutoyo 10x objective coupled to a 23µm-thick LSO scintillator. We used the side beam of BM05, which is produced by the last dipole of the synchrotron lattice before the short straight section that contains the dipole wiggler of BM05. This beam is partially superimposed with the main source of BM05, but on the side of the main beam. About 10mm of this beam with lower energy and higher coherence can be used for high-resolution imaging. It was filtered with 2.3mm of aluminum. The resulting detected average energy was 35keV. In total, 6000 projections of 40ms each were taken over 360 degrees, also in half-acquisition. The specimens were placed at a propagation distance of 180mm from the detector.

### Reconstruction and segmentation

µCT data was reconstructed into 8-bit BMP image stacks using NRecon version 1.6.10 (Bruker microCT, Belgium). Tomographic slices were reconstructed from PPC-SRµCT scans using filtered back-projection algorithm using the software PyHST2 (37) coupled with a modified version (38) of single-distance phase retrieval (39). The different sub-volumes were ring-corrected (40), vertically concatenated, converted to 16 bits, cropped using in-house developed Matlab systems, and saved in jpeg2000 format for a 10x file size reduction without loss of data precision. Delta/beta ratios used in the reconstruction were 500 for 0.727µm voxel size scans and 1000 for 3µm voxel-size scans.

BMP and jpeg2000 image stacks were imported into VGStudio MAX version 3.5.1 (Volume Graphics, Germany) for manual segmentation and rendering. Tissue volumes were measured using the Porosity/Inclusion Analysis module.

## Results

### DICE-PPC-SRµCT reveals histological information about mineralized and soft tissues

**Figure 1:**
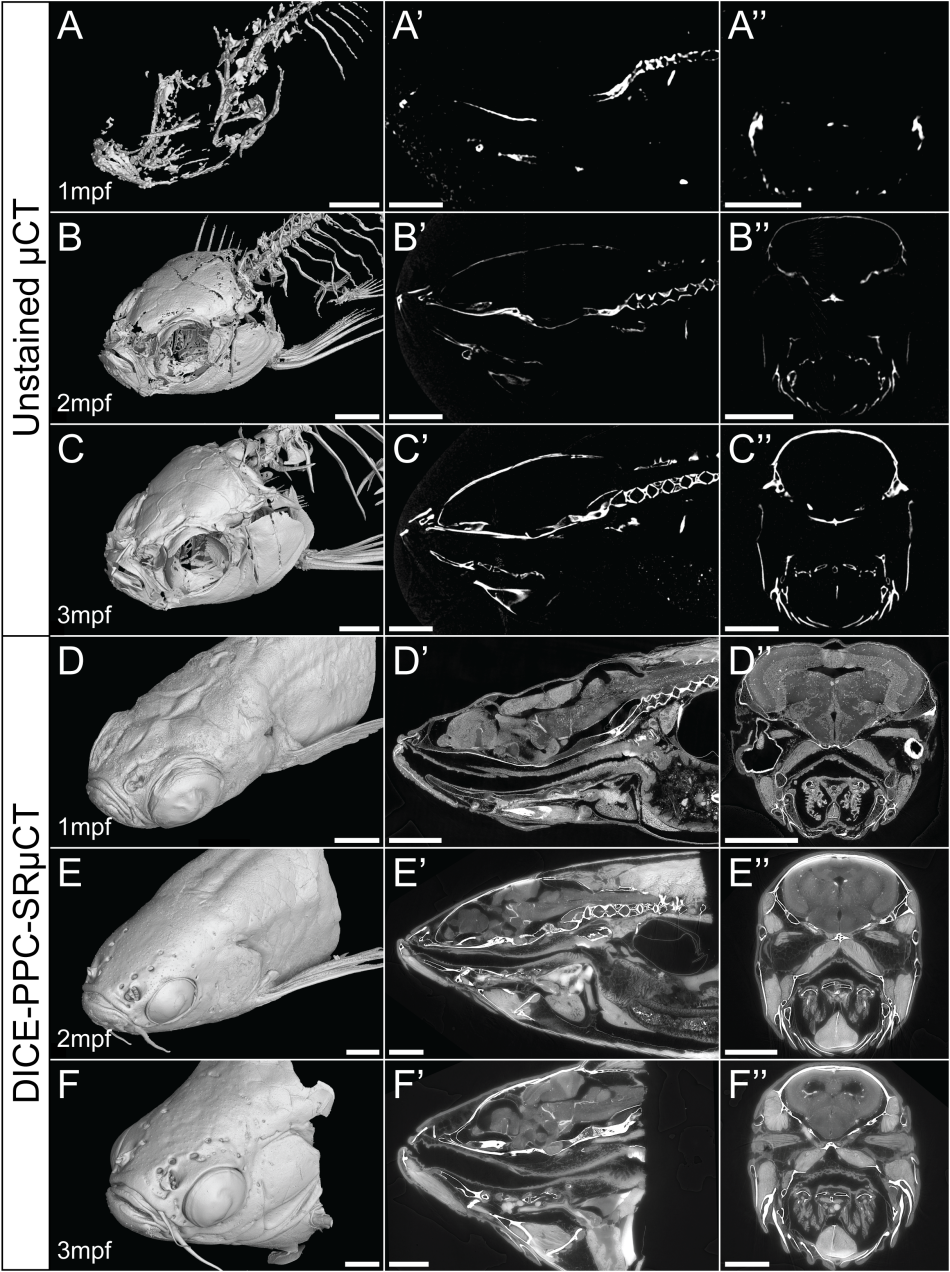
Conventional µCT only visualizes the zebrafish mineralized skeleton, while DICE-PPC-SRµCT adds soft-tissue information. **(A-C)** Volume renderings of 1-3mpf zebrafish scanned at a voxel size of 5.43µm using conventional µCT. **(D-F)** Volume renderings of 1-3mpf zebrafish stained with iodine and scanned at 0.727µm voxel size **(D)** and 3µm **(E, F)** using PPC-SRµCT. **(A’-F’)** Sagittal virtual thin sections through the midline. **(A’’-F’’)** Transverse virtual thin sections through the head (immediately posterior to the eyes). Scale bars, 500µm **(A-A’’, D-D’’)** and 1mm **(B-B’’, C-C’’, E-E’’, F-F’’)**.

Using conventional µCT without contrast enhancing agents can provide 3D data about the mineralized zebrafish skeleton, with the best results achieved in late juvenile or adults beyond 2-3mpf (**Figures 1B–C’’**). As the younger juvenile skeleton is only weakly mineralized and therefore less X-ray dense, fewer skeletal elements can be resolved (**Figures 1A–A’’**).

**Figure 2:**
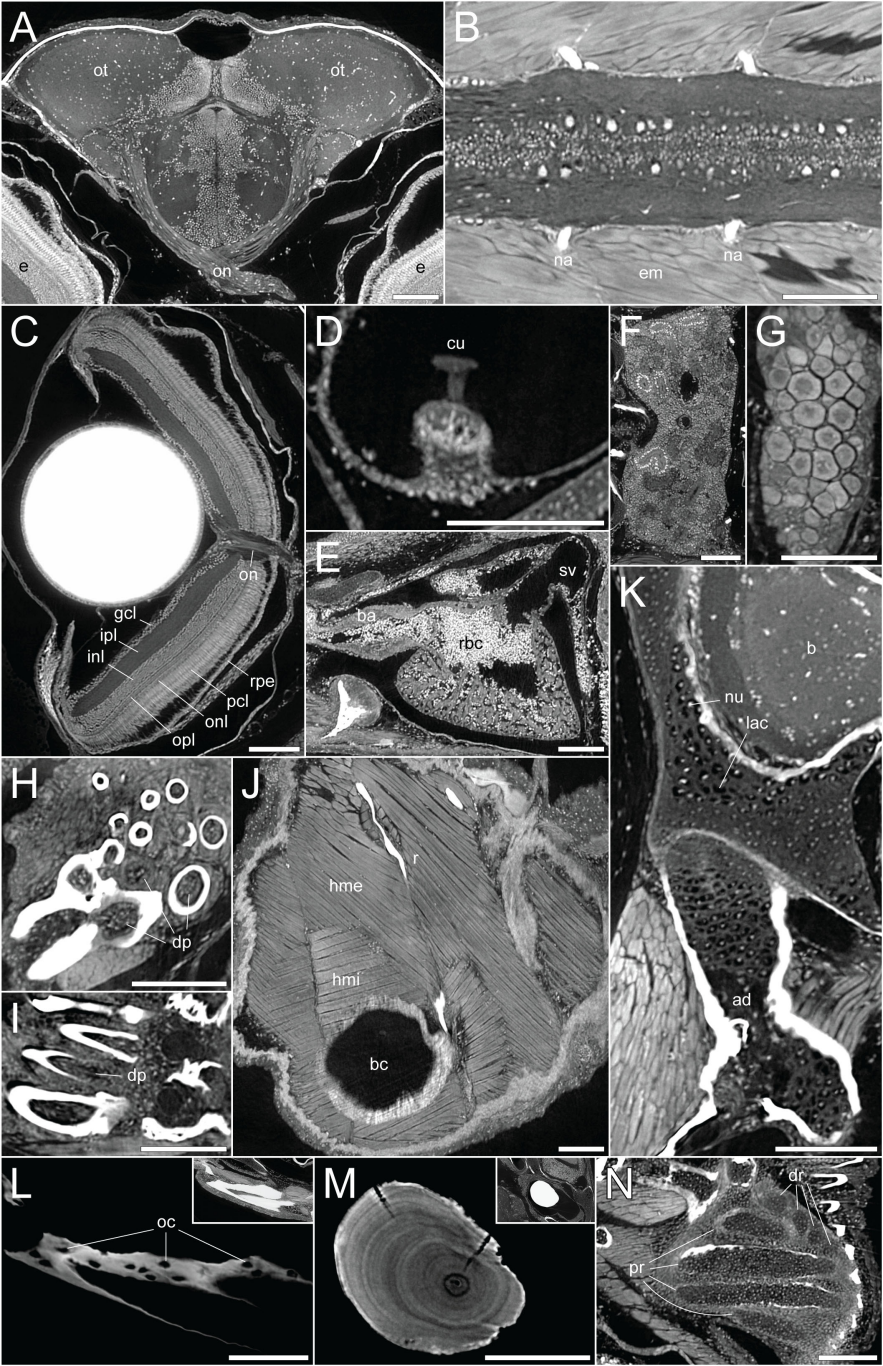
DICE-PPC-SRµCT at submicron resolution provides a detailed histological information. All images represent virtual thin sections from a 1mpf zebrafish scanned at 0.727µm voxel size. **(A)** Transverse view of the brain and optic nerves. **(B)** Longitudinal section through the spinal cord. **(C)** Transverse view of the eye, with the optic nerve and retinal layers visible. **(D)** Sagittal view of the crista ampullaris in the lateral semicircular canal. **(E)** Sagittal view of the heart. **(F)** Longitudinal view of the head region of the kidney. **(G)** Longitudinal view of the ovary. **(H, I)** Transverse and sagittal views of the pharyngeal teeth, with dental pulp visible. **(J)** Sagittal view of the two approximately perpendicular layers of hypaxial trunk musculature. **(K)** Sagittal view through the neurocranium (top) and hyomandibula (bottom), with visible chondrocyte lacunae and endochondral ossification of the hyomandibula. **(L)** Sagittal view through the dentary bone, using minimal projection with adjusted contrast to reveal osteocyte lacunae features in the bone. Inset image displays the same view but with comparable visualization settings to previous soft-tissue panels. **(M)** Sagittal section through the lapillus otolith, again with adjusted contrast to reveal the otolith microstructure showing growth rings. Inset image displays the same view but with comparable visualization settings to previous soft-tissue panels. **(N)** Section through the pectoral fin cartilaginous endoskeleton. ad, adipocytes; b, brain; ba, bulbus arteriosus; bc, body cavity; cu, cupula; dp, dental pulp; dr, distal radials; e, eye; em, epaxial muscle; gcl, ganglion cell layer; hme, hypaxial muscle (external); hmi, hypaxial muscle (internal); inl, inner nuclear layer; ipl, inner plexiform layer; lac, chondrocyte lacunae; na, neural arches; num, cell nuclei; oc, osteocytes; on, optic nerves; onl, outer nuclear layer; opl, outer plexiform layer; ot , optic tectum; pcl, photoreceptor cell layer; pr, proximal radials; r, rib; rbc, red blood cells; rpe, retinal pigmented epithelium; sv, sinus venosus. Scale bars, 100µm.

By employing DICE-PPC-SRµCT we could obtain scan data with down to submicron voxel sizes and near-histological quality already at 1mpf (**Figures 1D–F’’**; **Supplementary Videos 1**– **3**), allowing the visualization of range of soft tissues including the brain and spinal cord (**Figures 2A, B**), the retina (**Figure 2C**), the sensory organs in the semicircular canals (**Figure 2D**), organs such as the heart, kidney, and ovary (**Figures 2E–G**), teeth (**Figures 2H, I**), and trunk musculature (**Figure 2J**). Cartilage could be observed at the cellular level, with chondrocyte lacunae appearing as dark (unstained) cavities containing bright stained nuclei (**Figures 2K, N**). All this was achieved while simultaneously retaining the histological information in the dense, mineralized elements including bones and otoliths (**Figures 2L, M**).

**Figure 3:**
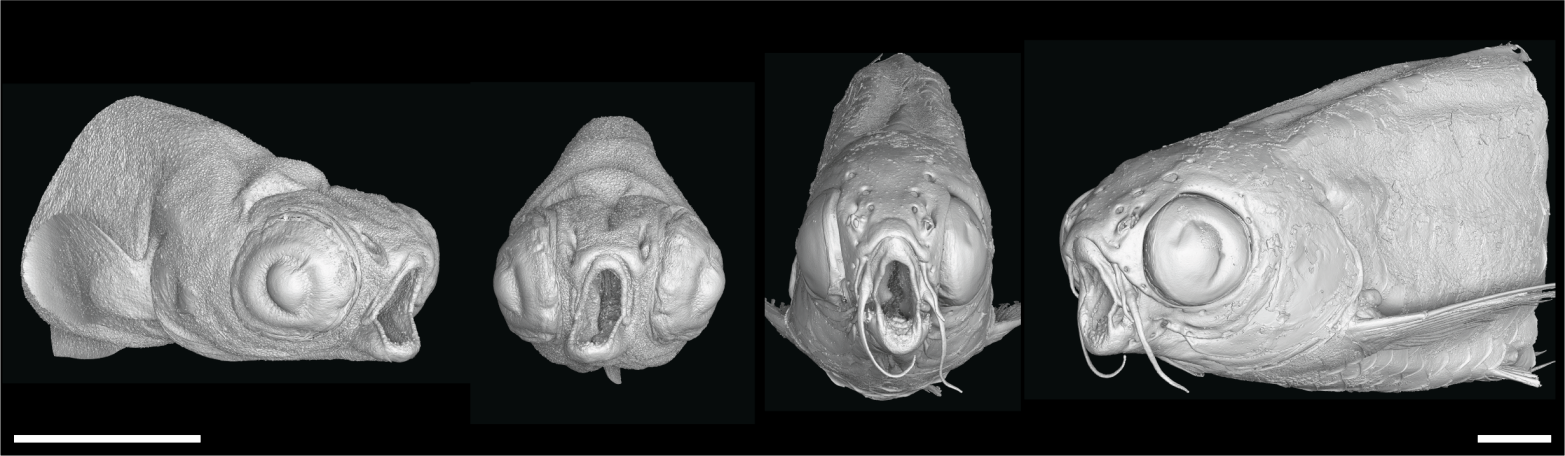
*nkx3.2* ^-/-^ zebrafish display the distinctive “open mouth” phenotype. Anterolateral and anterior volume renderings of DICE-PPC-SRµCT scanned *nkx3.2* ^-/-^ zebrafish at 1mpf (left) and 2mpf (right). Scale bars, 1mm.

To demonstrate how these datasets can be used to uncover skeletal-associated soft tissue phenotypes, we scanned two wild-type and two *nkx3.2* ^-/-^ mutant zebrafish at 1mpf (late larva; 0.727µm voxel size) and 2mpf (juvenile; 3µm voxel size). These phenotypes are most likely the secondary result of the primary skeletal deformities we previously described (34): the jaw joint fusion leading to the open-mouth phenotype (**Figure 3**) and the deformities of the occiput and Weberian apparatus.

### Identifying defects in jaw-associated musculature

**Figure 4:**
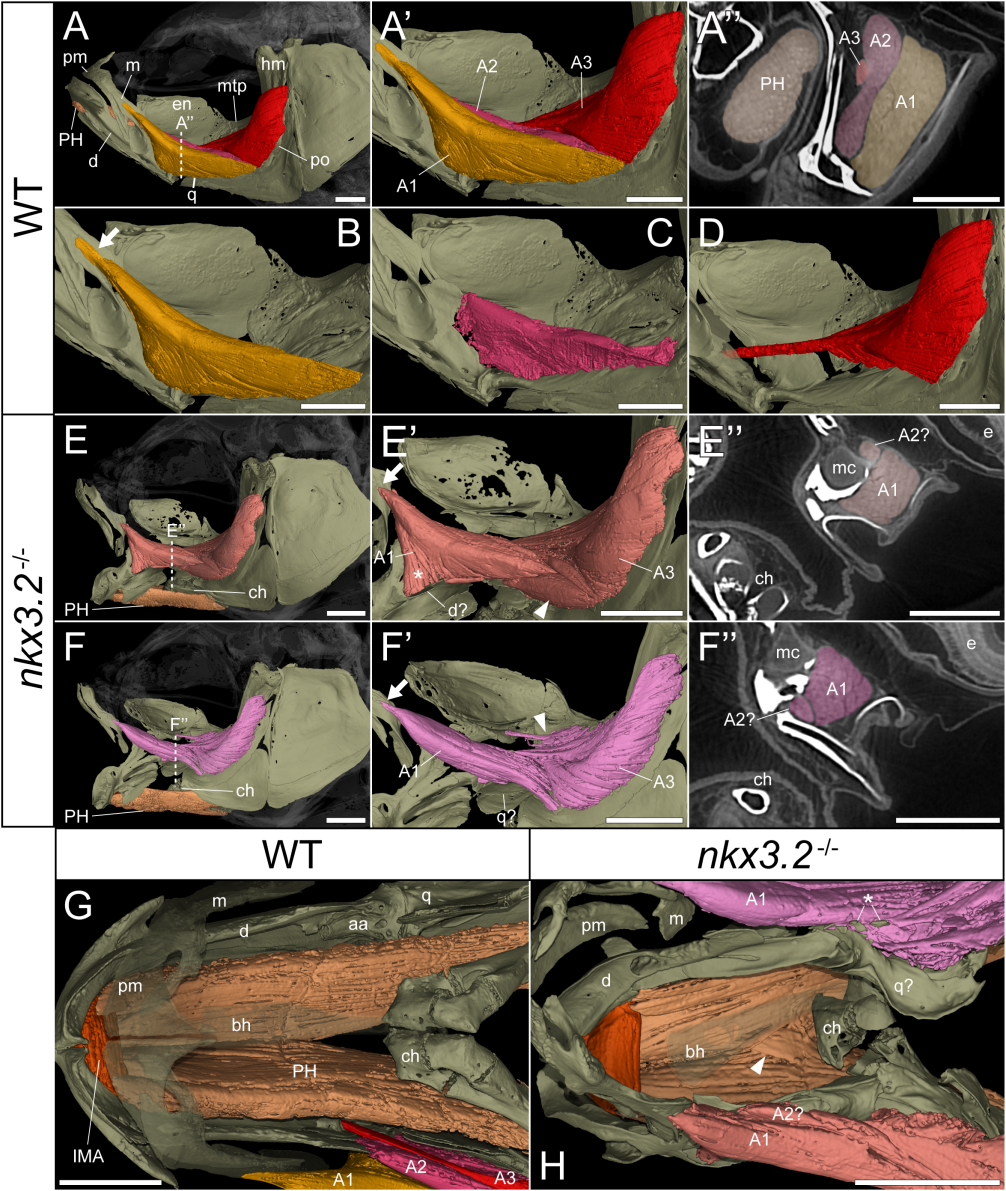
*nkx3.2* ^-/-^ zebrafish display defects in lower jaw-associated musculature. **(A)** Lateral volume rendering of the wild-type zebrafish head skeleton and relevant associated muscles. **(A’)** Close up of the rendered adductor mandibulae muscle bundles A1-A3. **(A’’)** False-coloured transverse virtual thin section [position indicated by dotted line in **(A)**] highlighting the protractor hyoideus muscle and distinct bundles of the adductor mandibulae. **(B–D)** Renderings of the isolated A1, A2, and A3 bundles of the adductor mandibulae, respectively. **(E, F)** The left and right lateral views of a single *nkx3.2* ^-/-^ zebrafish, with the panels in **(F-F’’)** reversed to facilitate comparison. Arrows in **(B, E’, F’)** indicate the maxillary tendon of A1. Asterisk in **(E’)** indicates the unusual anterior attachment site of the A1 bundle to the lower jaw. Arrowheads in **(E’)** and **(F’)** indicate the abnormal organization of muscle bundles. **(G, H)** Dorsal view of the lower jaw skeleton and associated musculature in wild-type and *nkx3.2* ^-/-^ fish, with the basihyal rendered transparent. In **(G)**, the maxilla and premaxilla were also rendered as partially transparent, and the right adductor mandibulae was removed to reveal the quadrate below. In **(H),** arrowhead indicates protractor hyoideus muscle fibers crossing the midline. Asterisk indicates ectopic ossifications embedded in the right adductor mandibulae. Both zebrafish are 2mpf and were scanned with a voxel size of 3µm using DICE-PPC-SRµCT. aa, anguloarticular; bh, basihyal; ch, ceratohyal; d, dentary; e, eye; en, endopterygoid; hm, hyomandibula; IMA, intermandibularis anterior; m, maxilla; mc, Meckel’s cartilage; mpt, metapterygoid; PH, protractor hyoideus; pm, premaxilla; po, preopercle; q, quadrate. All scale bars, 500µm, except on virtual thin sections **(A’’, E’’,** and **F’’)** 300µm.

As the jaw joint fusion characteristic of *nkx3.2* ^-/-^ mutants causes the lower jaw to be permanently locked in the open position (**Figure 3**), we chose to investigate whether this could have any secondary effects on the jaw-associated muscles at 2mpf. The zebrafish lower jaw is opened and closed primarily by two large muscles – the protractor hyoideus and adductor mandibulae, respectively (41, 42). The adductor mandibulae is divided into three main muscle bundles: A1, A2, and A3 (**Figures 4A–D**) (43), each with distinct attachment sites and functions that collectively serve to close the mouth. A1 is the maxillary component that has no connection to the mandible, instead connecting the maxilla to the quadrate and preopercle (**Figure 4B**) and functioning to retract the protruding upper jaw in concert as the rest of the adductor mandibulae complex retracts the mandible. A2 lies mesial to A1, connecting the posterior anguloarticular and dentary to the quadrate and preopercle (**Figures 4A’, C**). A3 is mesial to A2, and connects the coronomeckelian bone on the mesial surface of the dentary *via* a tendon to a broad muscle insertion on the preopercle, metapterygoid, and hyomandibula (**Figure 4D**).

In the *nkx3.2* ^-/-^ mutant, the adductor mandibulae complex appears poorly formed as A1, A2, and A3 are difficult to differentiate from each other compared to the wild-type appearance of three distinctive bundles (**Figures 4A’’, E’’, F’’**). Therefore, the 3D renderings of the mutant adductor mandibulae are displayed in single colors (**Figures 4E-F’’**) and external morphology was used to attempt to define the muscle bundles. The major components of A1 and A2 appeared to be present, as well as the broad muscle insertions of A3 in the preopercle and hyomandibula. However, the tendon connecting A3 to the mandible appeared to be absent or malformed. The mutant also displayed additional abnormal muscle bundles or tendinous extensions not found in the wild-type (**Figure 4E’, F’**, arrowheads).

In both the left and right adductor mandibulae of the *nkx3.2* ^-/-^ mutant, the maxillary tendinous insertion of A1 is present but appears considerably reduced in size compared to the wild-type (arrows in **Figures 4B, E’, F’**), while the ventral insertions appear different. In the absence of the jaw joint, the bones of the posterior dentary, anguloarticular, quadrate are disorganized and variably poorly shaped, making it difficult to assign definitive identities. However, the left A1 anteriormost ventral insertion is located unusually anteriorly in what would appear to be the posterior dentary (**Figure 4E’**, asterisk), while the right A1 anteriormost posteroventral insertion is located more posteriorly, in what is more likely to represent the quadrate (**Figure 4F’**), more similar to the wild-type condition.

The protractor hyoideus connects the anterior dentary to the ceratohyal and hypohyals, linking the first arch to the second arch (**Figure 4G**). Associated with the protractor hyoideus is the intermandibularis anterior, connecting the paired dentary bones (**Figure 4G**). In the *nkx3.2* ^-/-^ mutant these muscles appear relatively normal in terms of gross morphology and insertion sites, with the notable exception of a portion of muscle fibers of the protractor hyoideus crossing the midline division of this paired muscle (**Figure 4H**), likely related to the lateral distortions in the orientation of the ceratohyals and basihyal observed in this individual.

### Describing deformed and missing ligaments in the Weberian apparatus

**Figure 5:**
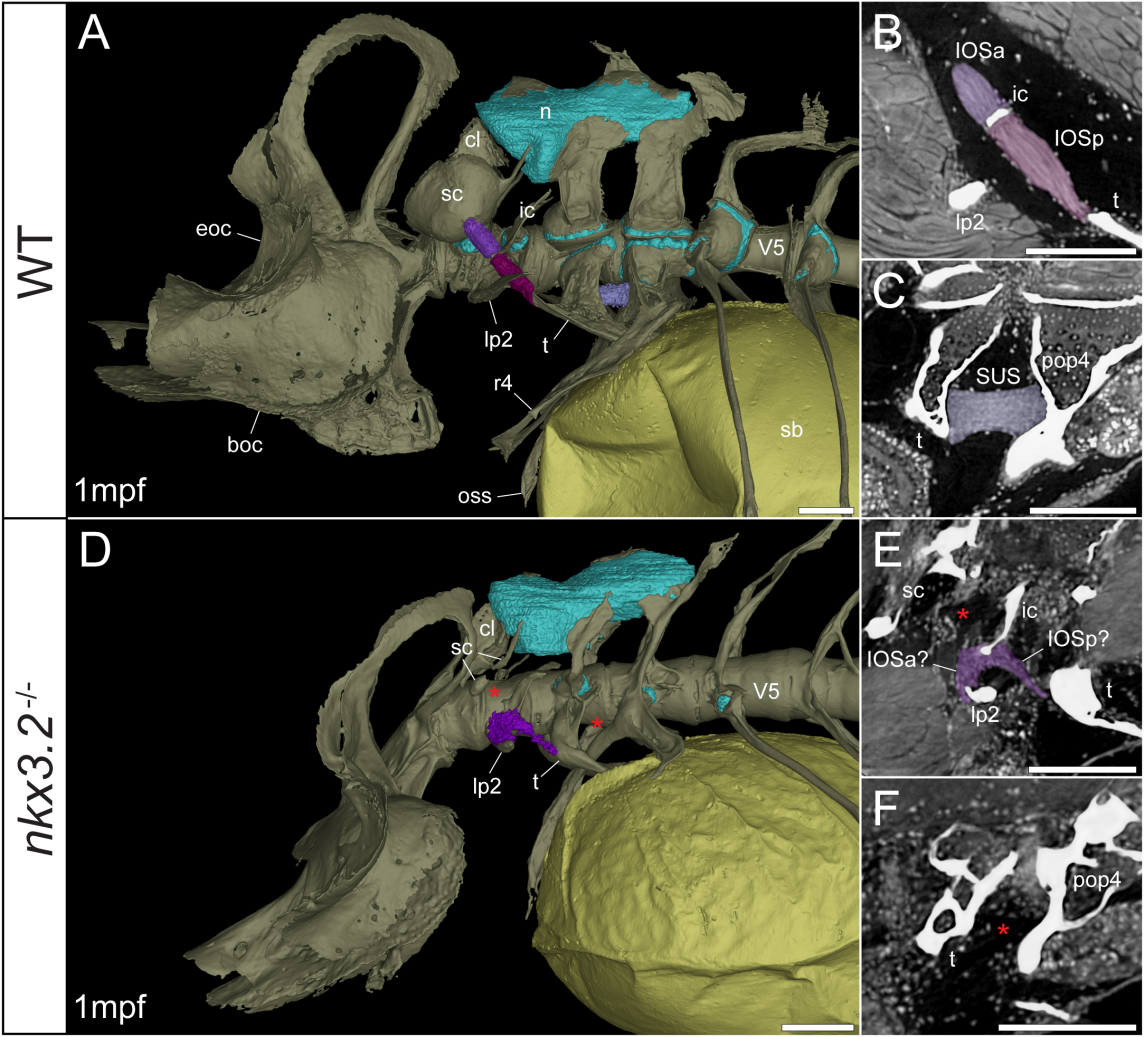
Late larval *nkx3.2* ^-/-^ zebrafish displays deformed and missing ligaments in the Weberian apparatus. **(A, D)** Lateral renderings of the occipital bones, Weberian apparatus, and anterior rib-bearing vertebrae (colored as bone) with associated cartilage (blue), ligaments (shades of purple), and swim bladder (yellow) of 1mpf wild-type and *nkx3.2* ^-/-^ zebrafish. Red asterisks in **(D)** indicate the absence of the anterior interossicular and suspensor ligaments. **(B, E)** False-coloured sagittal virtual thin sections through the interossicular ligament in wild-type and *nkx3.2* ^-/-^ zebrafish. Red asterisk in **(E)** indicates the absence of a ligamentous connection between the intercalarium and scaphium, as what may be anterior part of the interossicular ligament appears to be connected to lateral process instead. **(C, F)** False-coloured virtual thin sections through the suspensor ligament in wild-type and *nkx3.2* ^-/-^ zebrafish. The red asterisk in **(F)** indicates the absence of the suspensor ligament between the tripus and parapophysis 4. boc, basioccipital; cl, claustrum; eoc, exoccipital; ic, intercalarium; IOSa, anterior interossicular ligament; IOSp, posterior interossicular ligament; lp2, lateral process 2; n, neural complex cartilage; oss, os suspensorium; pop4, parapophysis 4; r4, rib 4; sb, swim bladder; sc, scaphium; SUS, suspensor ligament; t, tripus; V5, vertebrae 5. Scale bars, 100µm.

**Figure 6:**
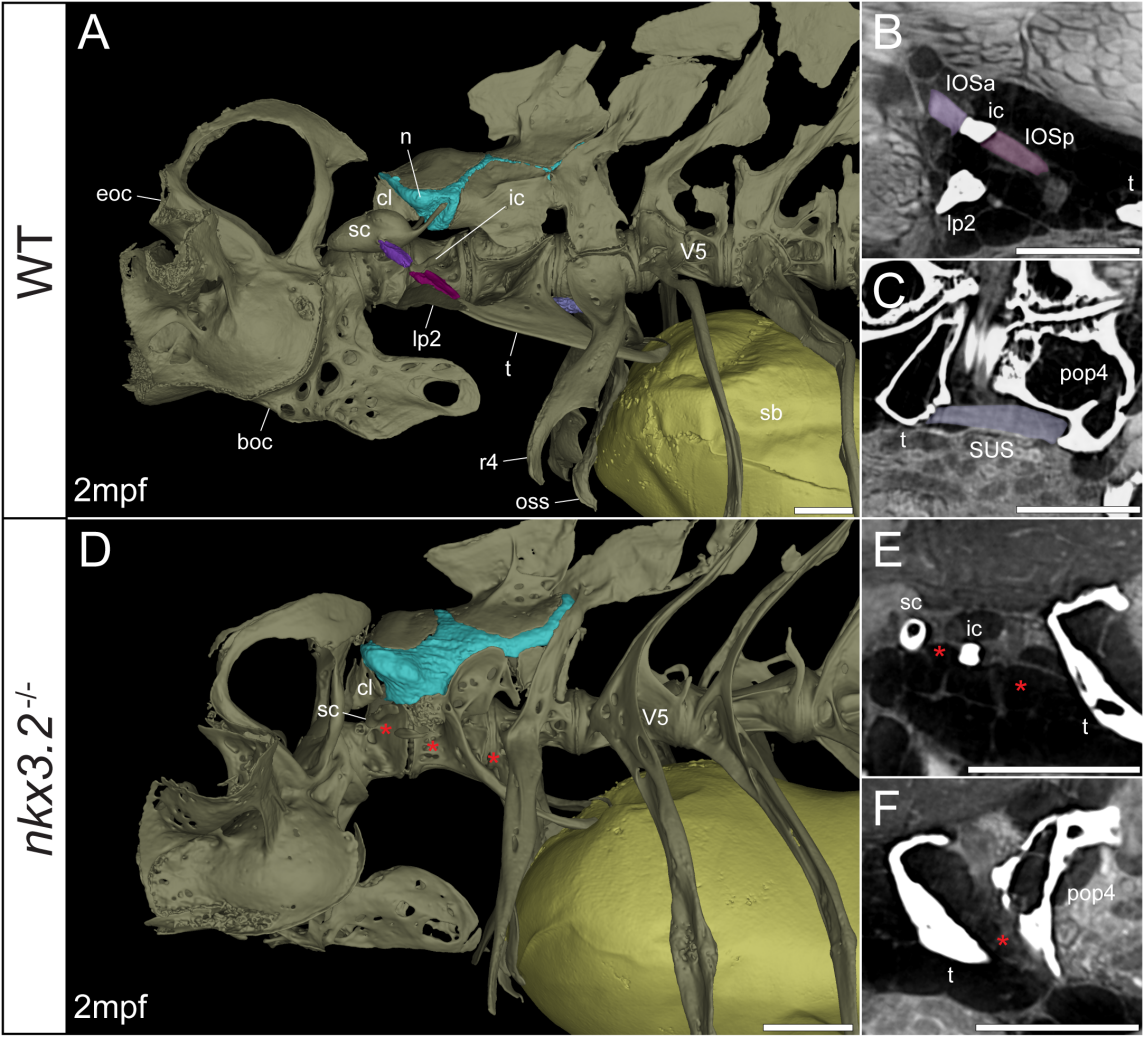
Juvenile *nkx3.2* ^-/-^ zebrafish displays missing ligaments in the Weberian apparatus. **(A, D)** Lateral renderings of the occipital bones, Weberian apparatus, and anterior rib-bearing vertebrae with associated cartilage (blue), ligaments (shades of purple), and swim bladder (yellow) of wild-type and *nkx3.2* ^-/-^ juvenile zebrafish. Red asterisks in **(D)** indicate the absence of the interossicular and suspensor ligaments. **(B, E)** False-coloured sagittal virtual thin sections through the interossicular ligament in wild-type and *nkx3.2* ^-/-^ zebrafish. Red asterisks in **(E)** indicate the absence of the interossicular ligament. **(C, F)** False-coloured virtual thin sections through the suspensor ligament in wild-type and *nkx3.2* ^-/-^ zebrafish. The red asterisk in **(F)** indicates the absence of the suspensor ligament between the tripus and parapophysis 4. boc, basioccipital; cl, claustrum; eoc, exoccipital; ic, intercalarium; IOSa, anterior interossicular ligament; IOSp, posterior interossicular ligament; lp2, lateral process 2; n, neural complex cartilage; oss, os suspensorium; pop4, parapophysis 4; r4, rib 4; sb, swim bladder; sc, scaphium; SUS, suspensor ligament; t, tripus; V5, vertebrae 5. Scale bars, 300µm.

The Weberian apparatus bones include vertebrae 1-4 and their modified ribs and neural arches (**Figures 5**, **6**) that facilitate the transmission of vibrations from the swim bladder into the inner ear (44–46). Cartilage (blue) is present at 1mpf in the endochondrally ossifying parapophyses of both the Weberian apparatus and rib-bearing vertebrae (**Figures 5A, C**), in addition to the dorsal neural complex that can be clearly seen at 1mpf (**Figure 5A**) before gradually being endochondrally ossified into the anterior supraneurals and neural arches (**Figure 6A**) (44, 47, 48).

Connecting the modified ventral bones are ligaments that collectively form a chain between the swim bladder and inner ear. The anterior surface of the swim bladder (tunica externa) is connected *via* a large ligament (“triple” ligament, 48) to both the posterior tripus and os suspensorium, associated with vertebrae 3 and 4, respectively. The thick suspensor ligament connects the parapophyses of vertebrae 3 (tripus) and 4. Finally, the interossicular ligament connects the anterior process of the tripus to the scaphium *via* the intercalarium midway between them, dividing the interossicular ligament into anterior and posterior bundles. This wild-type organization of the Weberian skeleton and interossicular and suspensor ligaments (shades of purple) in a late larva and juvenile are shown in **Figures 5A**, **6A**, along with sagittal virtual thin sections through the ligaments (**Figures 5B, C**, **6B, C**).

In the 1mpf mutant the neural complex cartilage appears normal (**Figure 5D**). However, there is no parapophyseal cartilage associated with vertebrae 1 or 2, and dramatically less cartilage present in the parapophyses of vertebrae 3 and 4, consistent with our previous description (34). However, this late larval mutant is unusual compared to previously characterized mutants in that it has a small cartilaginous parapophysis in the first rib-bearing vertebrae (V5; **Figure 5D**), while all other rib-bearing vertebrae have no parapophyses as previously described (34). Other than this, the skeletal defects of the tripus, lateral process 2, and scaphium (**Figure 5D**) are consistent with our previous description of the *nkx3.2* ^-/-^ mutant (34).

The swim bladder connection to the os suspensorium and posterior process of the tripus appears normal in the mutants, but the rest of the ligamentous auditory chain is highly disrupted (**Figures 5D**, **6D**). The suspensor ligament between the tripus and parapophysis 4 was entirely absent in both the 1mpf (**Figures 5D, F**) and 2mpf (**Figures 6D, F**) mutants. The interossicular ligament also appeared to be absent in the 2mpf mutant (**Figures 6D, E**), while the 1mpf mutant possessed only a small piece of connective tissue resembling a potential ligament weakly connecting the tripus to the intercalarium (posterior interossicular), and extending ventrally to attach the intercalarium to the underdeveloped lateral process 2 (**Figures 5D, E**), a connection not found in the wild-types (**Figures 5B**, **6B**).

### Characterizing altered structures of the inner ear

**Figure 7:**
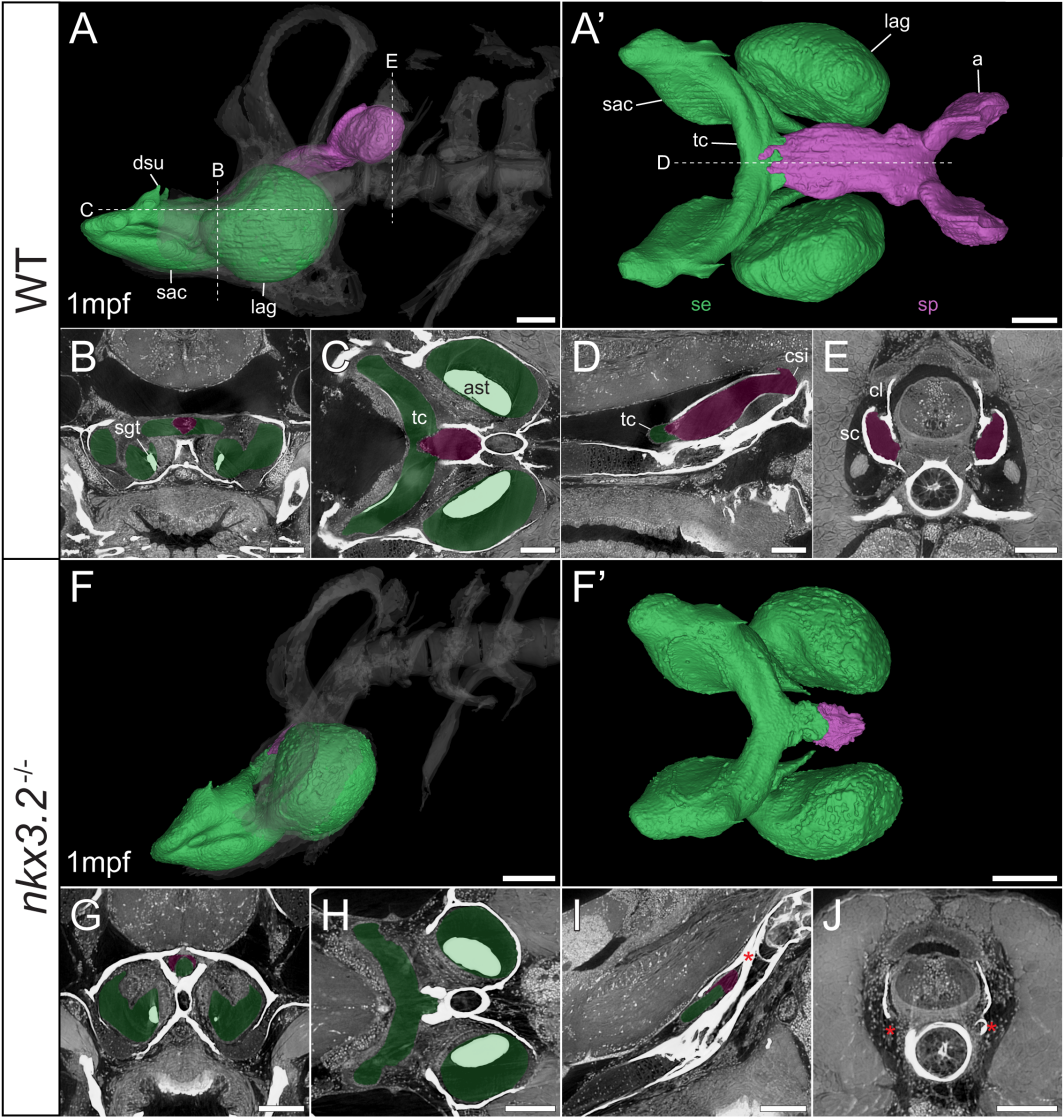
Late larval *nkx3.2* ^-/-^ zebrafish displays severe reduction of the sinus perilymphaticus. **(A, F)** Lateral rendering of the sinus endolymphaticus (green) and sinus perilymphaticus (magenta) in the context of the occiput and Weberian apparatus (white, transparent) in 1mpf wild-type and *nkx3.2* ^-/-^ zebrafish. **(A’, F’)** Anterodorsal renderings of the sinus endolymphaticus and sinus perilymphaticus (anterior to the left). False coloured transverse **(B, G)**, longitudinal **(C, H)**, and sagittal **(D, I)** virtual thin sections through the middle of the sinus endolymphaticus. Red asterisk in **(I)** indicates the blind posterior termination of the sinus impar. **(E, J)** False coloured transverse virtual thin sections through the atria of the sinus perilymphaticus. Red asterisks in **(J)** indicate the absence of the atria of the sinus perilymphaticus. Virtual thin section positions are indicated by labelled dotted lines in **(A, A’)**. a, atrium; ast, asteriscus; cl, claustrum; csi, cavum sinus impar; dsu, ductus sacculo-utricularis; lag, lagena; sac, saccule; sc, scaphium; se, sinus endolymphaticus; sgt, sagitta; sp, sinus perilymphaticus; tc, transverse canal. Scale bars, 100µm.

**Figure 8:**
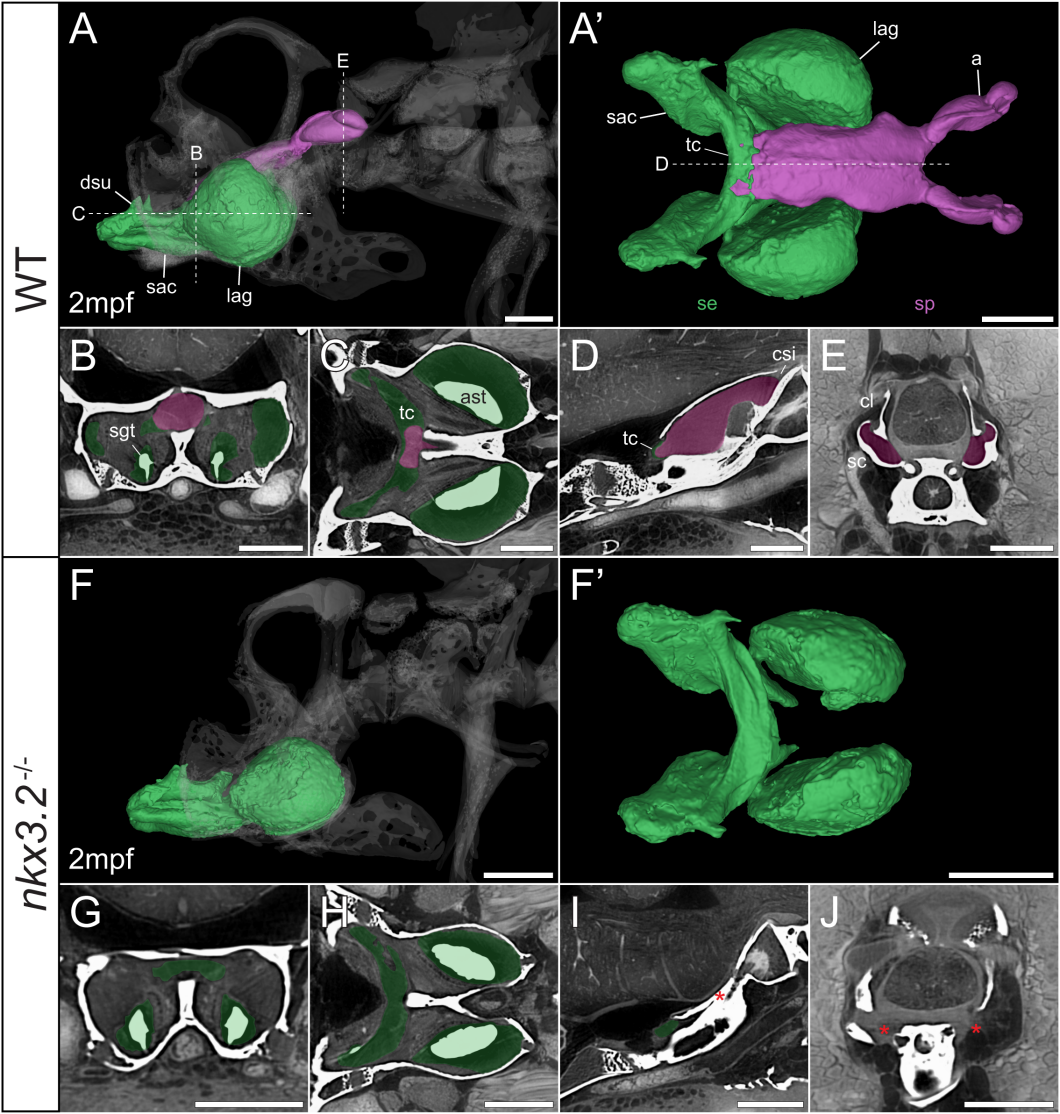
Juvenile *nkx3.2* ^-/-^ zebrafish displays complete absence of the sinus perilymphaticus. **(A, F)** Lateral rendering of the sinus endolymphaticus (green) and sinus perilymphaticus (magenta) in the context of the occiput and Weberian apparatus (white, transparent) in 2mpf wild-type and *nkx3.2* ^-/-^ zebrafish. **(A’, F’)** Anterodorsal renderings of the sinus endolymphaticus and sinus perilymphaticus (anterior to the left). **(B-D, G–I)** False coloured transverse, longitudinal, and sagittal virtual thin sections through the middle of the sinus endolymphaticus. Red asterisk in **(I)** indicates the blind posterior termination of the sinus impar. **(E, J)** False coloured transverse virtual thin sections through the atria of the sinus perilymphaticus. Red asterisks in **(J)** indicate the absence of the atria of the sinus perilymphaticus. Virtual thin section positions are indicated by labelled dotted lines in **(A, A’)**. a, atrium; ast, asteriscus; cl, claustrum; dsu, ductus sacculo-utricularis; lag, lagena; sac, saccule; sc, scaphium; se, sinus endolymphaticus; sgt, sagitta; sp, sinus perilymphaticus; tc, transverse canal. Scale bars, 300µm.

The mature zebrafish inner ear is comprised of the three semicircular canals with sensory cristae for vestibular function, and three end organs with sensory macula with a primarily auditory function (49, 50). Each of the three end organs, the utricle, saccule, and lagena, possess a single sensory macula covered by a dense calcium carbonate otolith. The semicircular canals and utricle make up the pars superior, while the saccule and lagena comprise the pars inferior. The pars superior and inferior are connected *via* the small ductus sacculo-utricularis (51, 52). All of these inner ear canals are filled with a shared endolymphatic fluid. In this description, we use the term “sinus endolymphaticus” (**Figures 7A, A’**, **8A, A’**) to refer to the space in the pars inferior that contains the endolymph.

As mentioned above, vibrations from the swim bladder travel anteriorly along the auditory chain until they reach the paired scaphia immediately posterior to the occiput (46). The gap between the scaphia and the sinus endolymphaticus of the inner ear is bridged by a second large sac containing perilymphatic fluid, the sinus perilymphaticus (also called sinus impar). The sinus perilymphaticus has paired atria that lie mesial to and partially surrounded by the cup-shaped scaphia, such that vibrations from the scaphia translate into motion of the perilymph (**Figures 7A, A’, E**, **8A, A’, E**). The paired atria extend anteriorly before merging and entering the occiput *via* the cavum sinus impar, an opening between the posterior exoccipital and basioccipital bones that lies dorsal to the notochord (**Figures 7D**, **8D**). The sinus perilymphaticus continues to extend anteriorly through the middle of the exoccipital, bounded ventrally by the basioccipital, until it reaches the midline of the transverse canal of the sinus endolymphaticus that connects the paired saccula. The perilymph and endolymph of these two compartments are separated by a thin membrane (52), but motion from the sinus perilymphaticus must be transmitted across this membrane to induce motion of the otoliths and stimulate the underlying sensory cells.

We segmented 3D models of the sinus endolymphaticus (green) and sinus perilymphaticus (magenta) in 1 and 2mpf wild-type zebrafish (**Figures 7A, A’**, **8A, A’**), and provide virtual thin sections that show the meeting point between these sacs (**Figures 7B–D**, **8B–D**) and how the atria of the sinus perilymphaticus relate to the scaphia and claustra (**Figures 7E**, **8E**). We previously described 3mpf *nkx3.2* ^-/-^ mutants displaying a closure/fusion of the cavum sinus impar between the exoccipital and basioccipital bones (34), and the same skeletal phenotype was observed in the 1 and 2mpf mutants included in this study. Here we extend this description by examining the effects of this phenotype on the sinus endolymphaticus and sinus perilymphaticus.

The 1mpf *nkx3.2* ^-/-^ mutant sinus perilymphaticus was extremely small and limited posteriorly (**Figures 7F, F’**), failing to extend out of the skull to reach the scaphia as a result of the closed cavum sinus impar (**Figure 7I**). The only indication that this small space posterior to the transverse canal actually represented the sinus perilymphaticus was the presence of a thin membrane separating itfrom the transverse cana (**Figures 7G, I**), too dorsal to be seen in **Figures 7H**. On the posterior side of the closed cavum sinus impar, nothing resembling a perilymphatic sac could be observed, and no atria were observed in association with the claustra or reduced scaphia (**Figure 7J**). The phenotype in the 2mpf mutant was almost identical, except the sinus perilymphaticus was absent entirely (**Figures 8F–J**). The sinus endolymphaticus, on the other hand, appeared quite normal in shape and extent in mutants (**Figures 7F, F’**, **8F, F’**).

### Volume measurements of 3D segmented soft tissues provide information about growth trajectories

**Figure 9:**
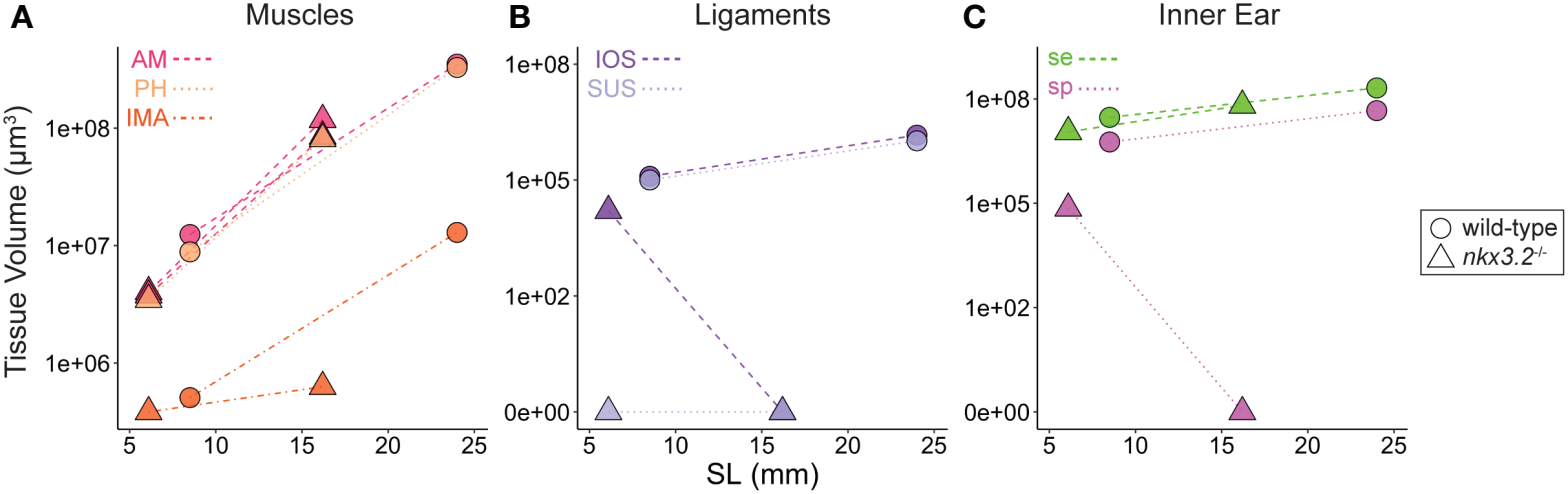
Volume measurements of 3D segmented soft tissues. Volume measurements of muscles **(A)**, Weberian ligaments **(B)**, and inner ear sacs **(C)**. Circles indicate wild-type tissues, while triangles indicate *nkx3.2* ^-/-^ tissues. Each point represents a single tissue volume measurement. Dotted lines connect corresponding tissues from 1mpf and 2mpf zebrafish of the same genotype to approximate growth trajectories. Different tissues are indicated by individual colors and line styles in agreement with the color codes used in the previous figures. Y-axes are log- or pseudolog-scaled. X-axes display standard length (mm). AM, adductor mandibulae; PH, protractor hyoideus; IMA, intermandibularis anterior; IOS, interossicular; SUS, suspensor; se, sinus endolymphaticus; sp, sinus perilymphaticus.

In addition to describing the shape of all of the above tissues qualitatively, the digital and high-resolution nature of the data makes it possible to acquire a wealth of additional quantitative data. Here, we used a simple measure, 3D volume, to describe the tissues and phenotypes. From the segmented volumes of the tissues displayed in the previous figures, precise volume measurements were performed and plotted in **Figure 9**. As zebrafish size can be variable even at identical ages, and our sample size was small, standard length was plotted against tissue volume in an attempt to enable to the comparison of approximate wild-type and mutant growth trajectories rather than comparing individuals.

By comparing the points and inferred growth trajectories of the wild-type and *nkx3.2* ^-/-^ mutants, it appears as though the adductor mandibulae and pars hyoideus muscles had their overall volumes relatively unchanged compared to the wild-type, while the intermandibularis anterior muscle volume was reduced (**Figure 9A**). The mutant Weberian ligaments are not as informative as only the 1mpf individual possessed just the interossicular ligament (the others being absent and given measured volumes of 0µm^3^), but it nevertheless reiterates that this ligament appears to be reduced in volume compared to what might be expected in a comparable wild-type individual (**Figure 9B**). Finally, the mutant sinus endolymphaticus appears approximately unchanged in volume relative to wild-types, or only slightly reduced, while the sinus perilymphaticus is clearly dramatically reduced as described qualitatively above.

All three categories of wild-type tissues appeared remarkably internally consistent in the relative tissue volumes, as the ratio between the volumes of the adductor mandibulae, pars hyoideus, and intermandibularis anterior were almost unchanged at 1mpf and 2mpf (parallel growth trajectories in **Figure 9A**), and the same was true for the interossicular and suspensor ligaments (**Figure 9B**) and the sinus endolymphaticus and sinus perilymphaticus (**Figure 9C**), suggesting that within each category, the tissues increase in size at comparable rates as the fish grows.

## Discussion

Whole-body histological examination in 3D is strongly desirable for characterization of organisms modeling skeletal diseases in order to carry out a rigorous comparison with human pathologies. Model organisms of small sizes like zebrafish currently display challenges in achieving high resolution datasets visualizing the interaction of the skeleton with the surrounding tissues. We applied diffusible iodine-based contrast enhancement (DICE) and propagation phase-contrast synchrotron radiation micro-computed tomography (PPC-SRµCT) to image late-larval and juvenile wild-type and *nkx3.2* ^-/-^ mutant zebrafish. The obtained datasets reveal high-quality, high-resolution virtual histological images of tissues and organs that can be segmented to get unprecedented 3D models of interaction between the mineralized skeleton and surrounding soft tissues.

In comparison, conventional µCT without a contrast enhancing agent provides little to no information about soft tissues. In addition, the best results for 3D segmentation of the bony skeleton are only achieved after 2 months post fertilization, because fewer skeletal elements can be resolved prior to a substantial degree of mineralization being completed. Iodine enhances contrast of soft tissues in addition to the mineralized skeleton (21, 24), whereas propagation phase contrast enhances the contrast at the edges of microstructural boundaries (25–27). The HiP-CT protocol of using a reference scan to reduce low-frequency background variation also further increases the soft-tissue contrast (36). Each of these three techniques independently enable the visualization of soft tissues in comparison to conventional µCT, and combined provide excellent image contrast for the relatively easy segmentation of the skeleton, soft tissues, and the boundaries between different tissue types. This can be advantageous for segmenting cartilage, muscle bundles, ligaments and tendons that are in direct contact with the mineralized skeleton.

By comparison, PTA enhances the contrast of soft tissues but does not seem to enhance the mineralized tissue (53, 54), leading to weaker contrast between mineralized and non-mineralized tissues and making it harder to segment the skeleton and soft tissues on the basis of apparent density (brightness in the image). If the 3D segmentation of the interaction between mineralized and soft tissues is not the focus of the examination, PTA and iodine are capable of producing comparable high quality histological data down to the cellular level using PPC-SRµCT (24, 54). Particularly for analyses of absolute rather than relative tissue volumes, it should also be noted that I_2_E causes shrinkage of many soft tissues due to the high concentration of ethanol, so other stains including PTA and pH-buffered I_2_KI could be more suitable (55–57).

Applying DICE-PPC-SRµCT to the zebrafish model for *spondylo-megaepiphyseal-metaphyseal dysplasia* represented by homozygous *nkx3.2* ^-/-^ mutants not only revealed the detailed morphology of the mutants with characteristic open mouth phenotypes but also uncovered changes in the muscle organization around the fused jaw joint. Importantly, we observed that the adductor mandibulae and protractor hyoideus muscles had disrupted fiber/bundle organization, and some force-transmitting tendons appeared reduced, possibly indicative of jaw muscle disuse in the mutants due to the inability to articulate the jaws (33, 58, 59). Particularly notable is the apparent insertion of one of the 2mpf A1 muscles to the dentary rather than the quadrate, crossing the fused jaw joint. This could indicate that the loss of the joint identity in the skeleton weakens the boundaries of the molecular patterning zones of the muscle attachment sites, suggesting these attachment sites can be labile in response to an altered underlying skeleton (60).

Surprisingly, the volume measurements of the jaw muscles revealed that both the adductor mandibulae and protractor hyoideus muscles in the mutants appeared to be consistent with the growth trajectories of the wild-type fish, suggesting overall muscle volumes were unaffected by the open jaw phenotype. On the other hand, the intermandibularis anterior muscle with apparently normal morphology and attachment sites displayed an apparent reduction in volume at 2mpf compared to the wild-type, which could indicate that this muscle is more sensitive to disuse than the adductor mandibulae and protractor hyoideus. Together, the morphological, histological, and volume information are important for the characterization of muscle pathologies in a malformed skeleton, although a greater number of *nkx3.2* ^-/-^ specimens would have to be analyzed to draw statistically significant quantitative conclusions in this case.

We previously hypothesized that sound transmission from the swim bladder to the inner ear would be severely negatively affected by the deformation of the Weberian ossicles and closure of the cavum sinus impar on the basis of µCT data from adult mutants (34). This additional data from late larval and juvenile mutants that reveals the severe reduction and/or loss of Weberian ligaments and the sinus perilymphaticus strongly support this conclusion, as almost every connection in the auditory chain from swim bladder to inner ear is now demonstrated to be disrupted or missing altogether. Even though the Weberian apparatus is a morphological novelty of otophysan fishes (45), understanding the relationship between malformed bones and connected ligaments and the interaction between the heavily affected sinus perilymphaticus and apparently unaffected sinus endolymphaticus could have important implications for understanding the phenotypic outcomes in human pathologies involving similar bone-associated tissues.

These soft tissue phenotypes, in addition to those described above in the jaw musculature, are likely to be secondary effects of the malformed cartilage and bone of the skeleton that are the direct result of the *nkx3.2* knockout, as *nkx3.2* expression has not been described in these specific soft tissues. Axial ligaments are derived from the sclerotome (61, 62), where *nkx3.2* is a key regulator (63, 64), so we cannot rule out a direct effect of *nkx3.2* on axial fibrous tissues *via* disruption to the sclerotome. *nkx3.2* expression has also been described in association with the anterior notochord in juvenile zebrafish, although not with enough detail to distinguish precise tissues such as cartilage or ligaments (65).

Our histopathological findings in bone-associated soft tissues obtained by applying DICE-PPC-SRµCT demonstrates a great potential of this technique for the investigation of small fish skeletal disease models involving gene mutants and regulatory sequence mutants that often display subtle phenotypes (66), and also for skeleton-associated regeneration experiments and general developmental studies of, for example, mineralization progression. For some questions, particularly those involving adult fish and large soft tissues, conventional µCT combined with contrasting agents may be sufficient, as has been already shown for mouse (67) and in a limited number of zebrafish studies (e.g. 22, 68). However, 3D characterization of larval and juvenile tissues or those that require histological detail at least for now most probably will rely on the phase contrast capabilities and characteristic brightness of large synchrotron light sources, although as technologies advance these properties may become more accessible in lab-based facilities (27, 69, 70).

DICE-PPC-SRµCT scans generate a large volume of data (tens to hundreds of Gbs per specimen), and a large number of specimens can be scanned at a relatively high-throughput. Therefore, and in addition to the limited accessibility of synchrotron facilities to most researchers, key limiting factors can be the time, hardware, and software resources required to analyze the scans and generate relevant biological data by manual segmentation. However, in parallel to advances in imaging in recent years, various machine-learning image analysis techniques have been developed that can be applied to these large, complex datasets to obtain reproducible quantitative information in a significantly reduced timeframe (e.g. 53, 71–74).

In summary, we describe a novel protocol for the DICE-PPC-SRµCT of zebrafish larvae and juveniles, and use the *nkx3.2* ^-/-^ mutant line to demonstrate how the generated datasets can be used for the detailed histopathological and 3D analysis of phenotypes in skeletal-associated soft tissues. We encourage other small-fish researchers and the developmental biology community as a whole with access to conventional µCT or synchrotron light sources to explore the processes of development, growth, aging, and regeneration with the benefit of high-quality 3D histological data of soft tissues.

## Data availability statement

The datasets presented in this study can be found in online repositories. The names of the repository/repositories and accession number(s) can be found below: https://data.esrf.fr/doi/10.15151/ESRF-DC-1057386678.

## Ethics statement

The animal study was reviewed and approved by the local ethics committee for animal research in Uppsala, Sweden (permit number 5.8.18-18096/2019).

## Author contributions

TH designed the study with contribution of SS and JL. Beamtime was granted through a successful application (LS3021) designed by SS with contribution from TH, JL, PT and KD. TH performed the staining. PT, KD, SS and JL performed the tomographic experiment. PT and KD reconstructed the data. JL segmented the data, and TH and JL analysed the data with contribution from SS. TH and JL wrote the manuscript, with contribution from SS, PT and KD. All authors contributed to the article and approved the submitted version.

## Supplementary material

Supplementary Video 1 Anterior to posterior transverse virtual thin sections through the wild-type 1mpf zebrafish obtained with DICE-PPC-SRµCT.

Supplementary Video 2 Lateral sagittal virtual thin sections through the wild-type 1mpf zebrafish obtained with DICE-PPC-SRµCT.

Supplementary Video 3 Dorsal-ventral longitudinal virtual thin sections through the wild-type 1mpf zebrafish obtained with DICE-PPC-SRµCT.

## References

[1] Zelzer E Olsen BR. The genetic basis for skeletal diseases. Nature (2003) 423:343–8. doi: 10.1038/nature01659 12748653

[2] Long F Ornitz DM. Development of the endochondral skeleton. Cold Spring Harb. Perspect Biol (2013) 5:a008334–a008334. doi: 10.1101/cshperspect.a008334 23284041 PMC3579395

[3] Veilleux L-N Trejo P Rauch F. Muscle abnormalities in osteogenesis imperfecta. J Musculoskelet Neuronal Interact (2017) 17:1–7. 28574406 PMC5492314

[4] Veilleux L-N Rauch F. Muscle-bone interactions in pediatric bone diseases. Curr Osteoporos. Rep (2017) 15:425–32. doi: 10.1007/s11914-017-0396-6 28856575

[5] Kapp-Simon KA Speltz ML Cunningham ML Patel PK Tomita T. Neurodevelopment of children with single suture craniosynostosis: A review. Childs Nerv. Syst (2007) 23:269–81. doi: 10.1007/s00381-006-0251-z 17186250

[6] Bonewald L. Use it or lose it to age: A review of bone and muscle communication. Bone (2019) 120:212–8. doi: 10.1016/j.bone.2018.11.002 PMC6360108 30408611

[7] Moutsopoulos HM Zampeli E. Bone, cartilage, and soft tissue disorders. In: Moutsopoulos HM Zampeli E, editors. Immunology and rheumatology in questions. Cham, Switzerland: Springer International Publishing, (2021) 175–87. doi: 10.1007/978-3-030-56670-8_12

[8] Dietrich K Fiedler IA Kurzyukova A López-Delgado AC McGowan LM Geurtzen K. Skeletal biology and disease modeling in zebrafish. J Bone Miner. Res (2021) 36:436–58. doi: 10.1002/jbmr.4256 33484578

[9] Marí-Beffa M Mesa-Román AB Duran I. Zebrafish models for human skeletal disorders. Front Genet (2021) 12:675331. doi: 10.3389/fgene.2021.675331 34490030 PMC8418114

[10] Howe K Clark MD Torroja CF Torrance J Berthelot C Muffato M. The zebrafish reference genome sequence and its relationship to the human genome. Nature (2013) 496:498–503. doi: 10.1038/nature12111 23594743 PMC3703927

[11] Howe DG Bradford YM Eagle A Fashena D Frazer K Kalita P. The zebrafish model organism database: new support for human disease models, mutation details, gene expression phenotypes and searching. Nucleic Acids Res (2017) 45:D758–68. doi: 10.1093/nar/gkw1116 PMC5210580 27899582

[12] Gonzales APW Yeh J-RJ. . Cas9-based genome editing in zebrafish. In: Doudna JA Sontheimer EJ, editors. Methods Enzymology 546:377–413. doi: 10.1016/B978-0-12-801185-0.00018-0 25398350

[13] Sharma P Sharma BS Verma RJ. CRISPR-based genome editing of zebrafish, in: Singh, vijai (Ed.), progress in molecular biology and translational science. Elsevier (2021) pp:69–84. doi: 10.1016/bs.pmbts.2021.01.005 33934838

[14] Mohideen M.-A.P.K. Beckwith LG Tsao-Wu GS Moore JL Wong ACC Chinoy MR. Histology-based screen for zebrafish mutants with abnormal cell differentiation. Dev Dyn. (2003) 228:414–23. doi: 10.1002/dvdy.10407 14579380

[15] Adissu HA Estabel J Sunter D Tuck E Hooks Y Carragher DM. Histopathology reveals correlative and unique phenotypes in a high throughput mouse phenotyping screen. Dis Model Mech (2014). 7 (5):515–24 doi: 10.1242/dmm.015263 PMC4007403 24652767

[16] Askary A Smeeton J Paul S Schindler S Braasch I Ellis NA. Ancient origin of lubricated joints in bony vertebrates. eLife (2016) 5:e16415. doi: 10.7554/eLife.16415 27434666 PMC4951194

[17] Copper JE Budgeon LR Foutz CA van Rossum DB Vanselow DJ Hubley MJ. Comparative analysis of fixation and embedding techniques for optimized histological preparation of zebrafish. Comp Biochem Physiol Part C Toxicol Pharmacol (2018) 208:38–46. doi: 10.1016/j.cbpc.2017.11.003 PMC5936644 29157956

[18] Misu A Yamanaka H Aramaki T Kondo S Skerrett IM Iovine MK. Two different functions of Connexin43 confer two different bone phenotypes in zebrafish. J Biol Chem (2016) 291:12601–11. doi: 10.1074/jbc.M116.720110 PMC4933477 27129238

[19] Charles JF Sury M Tsang K Urso K Henke K Huang Y. Utility of quantitative micro-computed tomographic analysis in zebrafish to define gene function during skeletogenesis. Bone (2017) 101:162–71. doi: 10.1016/j.bone.2017.05.001 PMC5512604 28476577

[20] Hur M Gistelinck CA Huber P Lee J Thompson MH Monstad-Rios AT. MicroCT-based phenomics in the zebrafish skeleton reveals virtues of deep phenotyping in a distributed organ system. eLife (2017) 6:e26014. doi: 10.7554/eLife.26014 28884682 PMC5606849

[21] Metscher BD. Micro CT for comparative morphology: Simple staining methods allow high-contrast 3D imaging of diverse non-mineralized animal tissues. BMC Physiol (2009) 9:11. doi: 10.1186/1472-6793-9-11 19545439 PMC2717911

[22] Babaei F Hong TLC Yeung K Cheng SH Lam YW. Contrast-enhanced X-ray micro-computed tomography as a versatile method for anatomical studies of adult zebrafish. Zebrafish (2016) 13:310–6. doi: 10.1089/zeb.2016.1245 27058023

[23] Gignac PM Kley NJ Clarke JA Colbert MW Morhardt AC Cerio D. Diffusible iodine-based contrast-enhanced computed tomography (diceCT): An emerging tool for rapid, high-resolution, 3-d imaging of metazoan soft tissues. J Anat. (2016) 228:889–909. doi: 10.1111/joa.12449 26970556 PMC5341577

[24] Khonsari RH Healy C Ohazama A Sharpe PT Dutel H Charles C. Submicron imaging of soft-tissues using low-dose phase-contrast X-ray synchrotron microtomography with an iodine contrast agent. Anat. Rec. (2014) 297:1803–7. doi: 10.1002/ar.22997 25044664

[25] Wilkins SW Gureyev TE Gao D Pogany A Stevenson AW. Phase-constrast imaging using polychromatic hard X-rays. Nature (1996) 384:335–8. doi: 10.1038/384335a0

[26] Cloetens P Pateyron-Salomé M Buffière JY Peix G Baruchel J Peyrin F. Observation of microstructure and damage in materials by phase sensitive radiography and tomography. J Appl Phys (1997) 81:5878–86. doi: 10.1063/1.364374

[27] Vågberg W Larsson DH Li M Arner A Hertz HM. X-Ray phase-contrast tomography for high-spatial-resolution zebrafish muscle imaging. Sci Rep (2015) 5:16625. doi: 10.1038/srep16625 26564785 PMC4643221

[28] Margaritondo G Hwu Y. Imaging with coherent X-rays: From the early synchrotron tests to SYNAPSE. J Imaging (2021) 7:132. doi: 10.3390/jimaging7080132 34460768 PMC8404945

[29] Tafforeau P Boistel R Boller E Bravin A Brunet M Chaimanee Y. Applications of X-ray synchrotron microtomography for non-destructive 3D studies of paleontological specimens. Appl Phys Mater Sci Process. (2006) 83:195–202. doi: 10.1007/s00339-006-3507-2

[30] Betz O Wegst U Weide D Heethoff M Helfen L Lee W-K. Imaging applications of synchrotron X-ray phase-contrast microtomography in biological morphology and biomaterials science. i. general aspects of the technique and its advantages in the analysis of millimetre-sized arthropod structure. J Microsc. (2007) 227:51–71. doi: 10.1111/j.1365-2818.2007.01785.x 17635659

[31] Hellemans J Simon M Dheedene A Alanay Y Mihci E Rifai L. Homozygous inactivating mutations in the NKX3-2 gene result in spondylo-Megaepiphyseal-Metaphyseal dysplasia. Am J Hum Genet (2009) 85:916–22. doi: 10.1016/j.ajhg.2009.11.005 PMC2790567 20004766

[32] Simsek-Kiper PO Kosukcu C Akgun-Dogan O Gocmen R Utine GE Soyer T. A novel NKX3-2 mutation associated with perinatal lethal phenotype of spondylo-megaepiphyseal-metaphyseal dysplasia in a neonate. Eur J Med Genet (2019) 62:21–6. doi: 10.1016/j.ejmg.2018.04.013 29704686

[33] Miyashita T Baddam P Smeeton J Oel AP Natarajan N Gordon B. nkx3.2 mutant zebrafish accommodate the jaw joint loss through a phenocopy of the head shapes of Paleozoic jawless fish. J Exp Biol (2020) 223:jeb216945. doi: 10.1242/jeb.216945 32527964 PMC10668335

[34] Waldmann L Leyhr J Zhang H Öhman-Mägi C Allalou A Haitina T. The broad role of Nkx3.2 in the development of the zebrafish axial skeleton. PloS One (2021) 16:e0255953. doi: 10.1371/journal.pone.0255953 34411150 PMC8376051

[35] Cloetens P Barrett R Baruchel J Guigay J-P Schlenker M. Phase objects in synchrotron radiation hard x-ray imaging. J Phys Appl Phys (1996) 29:133–46. doi: 10.1088/0022-3727/29/1/023

[36] Walsh CL Tafforeau P Wagner WL Jafree DJ Bellier A Werlein C. Imaging intact human organs with local resolution of cellular structures using hierarchical phase-contrast tomography. Nat Methods (2021) 18:1532–41. doi: 10.1038/s41592-021-01317-x PMC8648561 34737453

[37] Mirone A Brun E Gouillart E Tafforeau P Kieffer J. The PyHST2 hybrid distributed code for high speed tomographic reconstruction with iterative reconstruction and *a priori* knowledge capabilities. Nucl Instrum. Methods Phys Res Sect. B Beam Interact Mater At. (2014) 324:41–8. doi: 10.1016/j.nimb.2013.09.030

[38] Sanchez S Ahlberg PE Trinajstic KM Mirone A Tafforeau P. Three-dimensional synchrotron virtual paleohistology: A new insight into the world of fossil bone microstructures. Microsc. Microanal. (2012) 18:1095–105. doi: 10.1017/S1431927612001079 23026256

[39] Paganin D Mayo SC Gureyev TE Miller PR Wilkins SW. Simultaneous phase and amplitude extraction from a single defocused image of a homogeneous object. J Microsc. (2002) 206:33–40. doi: 10.1046/j.1365-2818.2002.01010.x 12000561

[40] Lyckegaard A Johnson G Tafforeau P. Correction of ring artifacts in X-ray tomographic images. Int J Tomogr. Stat (2011) 18:10.

[41] Diogo R Hinits Y Hughes SM. Development of mandibular, hyoid and hypobranchial muscles in the zebrafish: Homologies and evolution of these muscles within bony fishes and tetrapods. BMC Dev Biol (2008) 8 (24). doi: 10.1186/1471-213X-8-24 PMC2270811 18307809

[42] Tulenko FJ Currie P. The zebrafish in biomedical research. Elsevier (2020) pp:115–21. doi: 10.1016/B978-0-12-812431-4.00012-9

[43] Staab KL Hernandez LP. Development of the cypriniform protrusible jaw complex in *Danio rerio*: Constructional insights for evolution. J Morphol. (2010) 271:814–25. doi: 10.1002/jmor.10836 20235155

[44] Grande T Young B. The ontogeny and homology of the weberian apparatus in the zebrafish danio rerio (Ostariophysi: Cypriniformes). Zool J Linn Soc (2004) 140:241–54. doi: 10.1111/j.1096-3642.2003.00097.x

[45] Diogo R. Origin, evolution and homologies of the weberian apparatus: A new insight. Int J Morphol (2009) 27:333–54. doi: 10.4067/S0717-95022009000200008

[46] Schulz-Mirbach T Ladich F Mittone A Olbinado M Bravin A Maiditsch IP. Auditory chain reaction: Effects of sound pressure and particle motion on auditory structures in fishes. PloS One (2020) 15:e0230578. doi: 10.1371/journal.pone.0230578 32218605 PMC7100961

[47] Bird NC Mabee PM. Developmental morphology of the axial skeleton of the zebrafish, danio rerio (Ostariophysi: Cyprinidae). Dev Dyn. (2003) 228:337–57. doi: 10.1002/dvdy.10387 14579374

[48] Bird NC Richardson SS Abels JR. Histological development and integration of the zebrafish weberian apparatus. Dev Dyn. (2020) 249:998–1017. doi: 10.1002/dvdy.172 32243643

[49] Whitfield TT Riley BB Chiang M-Y Phillips B. Development of the zebrafish inner ear. Dev Dyn. (2002) 223:427–58. doi: 10.1002/dvdy.10073 11921334

[50] Abbas L Whitfield TT. The zebrafish inner ear. In: Perry SF Ekker M Farrell AP Brauner CJ, editors. Zebrafish, fish physiology. Adademic Press (2010). p. 123–71. doi: 10.1016/S1546-5098(10)02904-3

[51] Evans HM. A contribution to the anatomy and physiology of the air-bladder and weberian ossicles in cyprinidae. Proc R Soc Lond Ser B Contain. Pap. Biol Character (1925) 97:545–76. doi: 10.1098/rspb.1925.0018

[52] Bang PI Sewell WF Malicki JJ. Morphology and cell type heterogeneities of the inner ear epithelia in adult and juvenile zebrafish (Danio rerio). J Comp Neurol (2001) 438:173–90. doi: 10.1002/cne.1308 11536187

[53] Weinhardt V Shkarin R Wernet T Wittbrodt J Baumbach T Loosli F. Quantitative morphometric analysis of adult teleost fish by X-ray computed tomography. Sci Rep (2018) 8:16531. doi: 10.1038/s41598-018-34848-z 30410001 PMC6224569

[54] Ding Y Vanselow DJ Yakovlev MA Katz SR Lin AY Clark DP. Computational 3D histological phenotyping of whole zebrafish by X-ray histotomography. eLife (2019) 8:e44898. doi: 10.7554/eLife.44898 31063133 PMC6559789

[55] Buytaert J Goyens J De Greef D Aerts P Dirckx J. Volume shrinkage of bone, brain and muscle tissue in sample preparation for micro-CT and light sheet fluorescence microscopy (LSFM). Microsc. Microanal. (2014) 20:1208–17. doi: 10.1017/S1431927614001329 24963987

[56] Li Z Ketcham RA Yan F Maisano JA Clarke JA. Comparison and evaluation of the effectiveness of two approaches of diffusible iodine-based contrast-enhanced computed tomography (diceCT) for avian cephalic material: diceCT FOR AVIAN CEPHALIC MATERIAL. J Exp Zoolog. B Mol Dev Evol (2016) 326:352–62. doi: 10.1002/jez.b.22692 27511594

[57] Dawood Y Hagoort J Siadari BA Ruijter JM Gunst QD Lobe NHJ. Reducing soft-tissue shrinkage artefacts caused by staining with lugol’s solution. Sci Rep (2021) 11:19781. doi: 10.1038/s41598-021-99202-2 34611247 PMC8492742

[58] Subramanian A Kanzaki LF Galloway JL Schilling TF. Mechanical force regulates tendon extracellular matrix organization and tenocyte morphogenesis through TGFbeta signaling. eLife (2018) 7:e38069. doi: 10.7554/eLife.38069 30475205 PMC6345564

[59] Chen P Chen Z Mitchell C Gao J Chen L Wang A. Intramuscular injection of botox causes tendon atrophy by induction of senescence of tendon-derived stem cells. Stem Cell Res Ther (2021) 12:38. doi: 10.1186/s13287-020-02084-w 33413592 PMC7791643

[60] Hawkins MB Henke K Harris MP. Latent developmental potential to form limb-like skeletal structures in zebrafish. Cell (2021) 184:899–911.e13. doi: 10.1016/j.cell.2021.01.003 33545089

[61] Williams S Alkhatib B Serra R. Development of the axial skeleton and intervertebral disc. In: Olsen BR, editor. Current topics in developmental biology, vol. pp . Elsevier (2019). p. 49–90. doi: 10.1016/bs.ctdb.2018.11.018 PMC6800124 30902259

[62] Bagnat M Gray RS. Development of a straight vertebrate body axis. Development (2020) 147:dev175794. doi: 10.1242/dev.175794 33023886 PMC7561478

[63] Rodrigo I Hill RE Balling R Münsterberg A Imai K. Pax1 and Pax9 activate Bapx1 to induce chondrogenic differentation in the sclerotome. Development (2003) 130:473–82. doi: 10.1242/dev.00240 12490554

[64] Ma RC Jacobs CT Sharma P Kocha KM Huang P. Stereotypic generation of axial tenocytes from bipartite sclerotome domains in zebrafish. PloS Genet (2018) 14:e1007775. doi: 10.1371/journal.pgen.1007775 30388110 PMC6235400

[65] Crotwell PL Mabee PM. Gene expression patterns underlying proximal-distal skeletal segmentation in late-stage zebrafish, danio rerio. Dev Dyn. (2007) 236:3111–28. doi: 10.1002/dvdy.21352 17948314

[66] Leyhr J Waldmann L Filipek-Górniok B Zhang H Allalou A Haitina T. A novel cis-regulatory element drives early expression of Nkx3.2 in the gnathostome primary jaw joint. eLife (2022) 11:e75749. doi: 10.7554/eLife.75749 36377467 PMC9665848

[67] Handschuh S Glösmann M. Mouse embryo phenotyping using X-ray microCT. Front Cell Dev Biol (2022) 10:949184. doi: 10.3389/fcell.2022.949184 36187491 PMC9523164

[68] Dimitriadi A Beis D Arvanitidis C Adriaens D Koumoundouros G. Developmental temperature has persistent, sexually dimorphic effects on zebrafish cardiac anatomy. Sci Rep (2018) 8:8125. doi: 10.1038/s41598-018-25991-8 29802254 PMC5970236

[69] Hornberger B Kasahara J Gifford M Ruth R Loewen R. A compact light source providing high-flux, quasi-monochromatic, tunable X-rays in the laboratory. In: Murokh A Spiga D, editors. Advances in laboratory-based X-ray sources, optics, and applications VII. presented at the advances in laboratory-based X-ray sources, optics, and applications VII, vol. p . San Diego, United States: SPIE (2019). p. 2. doi: 10.1117/12.2527356

[70] Huang J Günther B Achterhold K Cui Y Gleich B Dierolf M. Energy-dispersive X-ray absorption spectroscopy with an inverse Compton source. Sci Rep (2020) 10:8772. doi: 10.1038/s41598-020-65225-4 32472032 PMC7260230

[71] Lösel PD van de Kamp T Jayme A Ershov A Faragó T Pichler O. Introducing biomedisa as an open-source online platform for biomedical image segmentation. Nat Commun (2020) 11:5577. doi: 10.1038/s41467-020-19303-w 33149150 PMC7642381

[72] Arzt M Deschamps J Schmied C Pietzsch T Schmidt D Tomancak P. LABKIT: Labeling and segmentation toolkit for big image data. Front Comput Sci (2022) 4:777728. doi: 10.3389/fcomp.2022.777728

[73] Lösel PD Monchanin C Lebrun R Jayme A Relle J Devaud J-M. Natural variability in bee brain size and symmetry revealed by micro-CT imaging and deep learning. bioRxiv (2022). doi: 10.1101/2022.10.12.511944 PMC10569549 37782674

[74] Wang A Zhang Q Han Y Megason S Hormoz S Mosaliganti KR. A novel deep learning-based 3D cell segmentation framework for future image-based disease detection. Sci Rep (2022) 12:342. doi: 10.1038/s41598-021-04048-3 35013443 PMC8748745

